# The ethics of AI-assisted warfighter enhancement research and experimentation: Historical perspectives and ethical challenges

**DOI:** 10.3389/fdata.2022.978734

**Published:** 2022-09-09

**Authors:** Jonathan Moreno, Michael L. Gross, Jack Becker, Blake Hereth, Neil D. Shortland, Nicholas G. Evans

**Affiliations:** ^1^Department of Bioethics, School of Medicine, University of Pennsylvania, Philadelphia, PA, United States; ^2^School of Political Science, University of Haifa, Haifa, Israel; ^3^Harvard Law School, Cambridge, MA, United States; ^4^Department of Philosophy, University of Massachusetts at Lowell, Lowell, MA, United States; ^5^School of Criminology and Justice Studies, University of Massachusetts at Lowell, Lowell, MA, United States

**Keywords:** artificial intelligence, warfighter enhancement, human research, experimentation, super soldiers

## Abstract

The military applications of AI raise myriad ethical challenges. Critical among them is how AI integrates with human decision making to enhance cognitive performance on the battlefield. AI applications range from augmented reality devices to assist learning and improve training to implantable Brain-Computer Interfaces (BCI) to create bionic “super soldiers.” As these technologies mature, AI-wired warfighters face potential affronts to cognitive liberty, psychological and physiological health risks and obstacles to integrating into military and civil society during their service and upon discharge. Before coming online and operational, however, AI-assisted technologies and neural interfaces require extensive research and human experimentation. Each endeavor raises additional ethical concerns that have been historically ignored thereby leaving military and medical scientists without a cogent ethics protocol for sustainable research. In this way, this paper is a “prequel” to the current debate over enhancement which largely considers neuro-technologies once they are already out the door and operational. To lay the ethics foundation for AI-assisted warfighter enhancement research, we present an historical overview of its technological development followed by a presentation of salient ethics research issues (ICRC, 2006). We begin with a historical survey of AI neuro-enhancement research highlighting the ethics lacunae of its development. We demonstrate the unique ethical problems posed by the convergence of several technologies in the military research setting. Then we address these deficiencies by emphasizing how AI-assisted warfighter enhancement research must pay particular attention to military necessity, and the medical and military cost-benefit tradeoffs of emerging technologies, all attending to the unique status of warfighters as experimental subjects. Finally, our focus is the enhancement of friendly or compatriot warfighters and not, as others have focused, enhancements intended to pacify enemy warfighters.

## Introduction

Since the turn of the century, the ethics of research on human performance enhancement in the civilian setting has become an area of vigorous scholarship, not only with regard to compliance with traditional ethical standards but also in light of developments in related fields like cognitive neuroscience that seeks to understand the structure of the human brain and cognition; and artificial intelligence (AI) that seeks to develop machines capable of performing tasks that would ordinarily require human cognition. These fields have promise to enhance human capacities and improve performance in a range of tasks, such as through the use of brain-computer interfaces (BCI) that connect humans to computers, potentially in both directions, and even brain-to-brain interfaces. These fields, moreover, are interrelated: Neuroscience benefits greatly from artificial intelligence to probe the human brain and create novel technologies to investigate and treat disease or enhance performance. For instance, applications include emotion suppression, enhanced awareness, WiFi capability, and the like. AI, meanwhile, benefits from an understanding of human cognition and neurology to develop better and “smarter” machines capable of acting autonomously. These convergent fields are particularly attractive to, for example, the defense industry, for the ability to combine the lateral thinking and instinct of warfighters with the processing power of AI.

The military applications of AI raise myriad ethical challenges across countries [e.g., (Australian DoD (Department of Defence), 2020; UK Ministry of Defence, 2021)]. Critical among them is how AI integrates with human decision making to enhance cognitive performance on the battlefield. AI applications range from augmented reality devices to assist learning and improve training to implantable BCI to create bionic “super soldiers.” As these technologies mature, AI-wired warfighters face potential affronts to cognitive liberty, psychological and physiological health risks and obstacles to integrating into military and civil society during their service and upon discharge (Denning et al., 2009). Before coming online and operational, however, AI-assisted technologies and neural interfaces require extensive research and human experimentation. Each endeavor raises additional ethical concerns that have been historically ignored thereby leaving military and medical scientists without a cogent ethics protocol for sustainable research. In this way, this paper is a “prequel” to the current debate over enhancement which largely considers neuro-technologies once they are already out the door and operational (ICRC, 2006).

To lay the ethics foundation for AI-assisted warfighter enhancement research, we present an historical overview of its technological development followed by a presentation of salient ethics research issues. We begin with a historical survey of AI neuro-enhancement research highlighting the ethics lacunae of its development. We demonstrate the unique ethical problems posed by the convergence of several technologies in the military research setting. Then we address these deficiencies by emphasizing how AI-assisted warfighter enhancement research must pay particular attention to military necessity, and the medical and military cost-benefit tradeoffs of emerging technologies, all attending to the unique status of warfighters as experimental subjects. Finally, our focus is the enhancement of friendly or compatriot warfighters and not, as others have emphasized (Hereth, 2022), enhancements intended to pacify enemy warfighters.

## Historical background of military artificial intelligence and neurotechnology

### Artificial intelligence and defense planning

In 1956, computer scientist John McCarthy organized the Dartmouth Summer Research Conference where the term “artificial intelligence” was coined. McCarthy was frustrated that little had been written about the idea that computers could possess intelligence. The 1956 Dartmouth conference is regarded as the origin of the approach known affectionately, and sometimes critically, as “good old-fashioned AI” or GOFAI, which is built on symbolic reasoning and logic. The more recent framework that utilizes mathematical models or “neural networks” capable of searching for patterns in vast quantities of data is often called “connectionism” and produces machine-learning using algorithms. Despite its rich history and ubiquity in the modern world, there remain certain basic disagreements both about what “AI” really means and whether advances in computing will ever lead to human-level intelligence or even a “superintelligence” that threatens human civilization. These disagreements about the definition and ultimate power of AI do not pose problems for this paper as our focus is on AI-*enabled* technologies, i.e., those that exploit systems that are generally regarded as based on principles of AI.

What can be said is that the Dartmouth conference established a fundamental assumption about the nature of intelligence itself, as a set of cognitive capacities directed toward problem-solving: thus any “artificial” intelligence would also be directed at problem-solving. That has set the tone for the goals of AI in all its multifarious applications. But intelligence is not only of the problem-solving variety; it also manifests itself in social and emotional contexts, for example. The tacit judgment required in those other contexts and exercised continuously by cognitively competent mature human beings has so far not been modeled in machines. Lacking what some logicians loosely call “intuition,” it is not at all clear that AI can achieve the most ambitious (and perhaps perilous) milestones often attributed to it^1^. In the military setting, the outstanding question is whether AI can not only reliably contribute to strategic goals and tactical planning but is also effective at the operational level.

As this debate has evolved in the past decade, US defense planners have de-emphasized general AI and fully autonomous systems as a goal, perhaps partly in response to worries about a “doomsday device” with no human interruption possible, thus becoming too similar to an automatic weapon. In 2016, speaking of the US government's new doctrine for asymmetric advantage or “offset” over potential adversaries, Deputy Defense Secretary Robert Work remarked that “people say ‘what's the Third Offset Strategy^2^ about? And they say ‘oh, it's about AI and autonomy.' We say no… It's about human-machine collaborative combat networks.” While the reference to collaboration is reassuring, collaboration does not imply that absolute human control is always required. US Department of Defense directive 3000.09 on Autonomy in Weapon Systems currently requires that all systems “allow commanders and operators to exercise appropriate levels of human judgment over the use of force.” In the absence of any system capable of general AI computer scientists focus on narrow AI, systems that can perform specific tasks for which they were trained, like the systems for playing complex games like chess and Go. The limits of narrow AI raise questions about hacking and other technical measures that may interfere with warfighter operations. Flaws in the algorithms that run narrow AI systems also raise ethical issues, as in the cases of racial and gender bias. Apart from an adversary's disruptive measures and biased coding, research and development of AI-enabled technology with warfighters itself poses ethical challenges that brain-computer interfaces (BCI) exemplify.

### The emergence of AI-enabled brain-computer interfaces

BCI is a paradigmatic example of neurotechnology, understood as any technology that helps to influence and understand the brain and its functions. “A BCI is a computer-based system that acquires brain signals, analyzes them, and translates them into commands to an output device to carry out a desired action.” Those signals are able to control cursors, prostheses, wheelchairs and other devices. “True” BCI systems use only signals from the central nervous system (CNS) and not from peripheral muscle nerves. In general, brain signal acquisition can be accomplished in two ways. Scalp-recorded EEG signals (eBCI) and wearable augmented realty (AR) systems are non-invasive (Portillo-Lara et al., 2021, p. 3). In contrast, intracortical microarrays (iBCI) vary from semi-invasive neural technologies, such as electrocorticography (ECoG) that require a craniotomy to place epidural or subdural electrodes on the surface of the cortex, to deeply embedded intracortical BCI or ocular or auditory implants.

These techniques have offsetting advantages and disadvantages. An eBCI is non-invasive but signal acquisition through the skull and scalp is difficult, whereas iBCI may improve signal strength but requires surgery and its attendant risks. Conventional improvements in BCI-based devices will function as therapeutic interventions, e.g., controlling prosthetics to restore capacity, including restoring nervous system feedback through artificial limbs. However, these devices can also maintain and enhance human performance during training and deployment. What is not settled, however, are the conditions under which these performance enhancements ought to be tested on or used by warfighters.

BCI predates AI by decades but can operate under GOFAI or the newer connectionist models. In the 1920's, the University of Jena's Hans Berger demonstrated the ability to read out electrical activity in the human brain *via* electroencephalography (EEG). The evolution of these fields illustrate how AI and BCI^3^ have converged thanks to innovations in reading the brain's electrical impulses^4^. In 1965, UCLA's Thelma Estrin articulated the requirements for a signal conversation system such that brain signals could be “digitized, filtered, classified and translated into cursor movement, for example, at very high speed.” These were in effect the requirements for a BCI^5^. Also at UCLA, “direct brain-computer communication” was outlined by Vidal (1973). In the words of one history:

“…the subject's EEG was to be transmitted to an amplifier the size of an entire desk belonging to the control area, which comprised two other screens and a printer. Then, after several steps, including analog-digital conversion, the signal would enter the IBM 360/91 for computing. Vidal asked, ‘Can these observable electrical brain signals be put to work as carriers of information in man-computer communication or for the purpose of controlling such external apparatus as prosthetic devices or spaceships?' And he answered, 'Even on the sole basis of the present states of the art of computer science and neurophysiology, one may suggest that such a feat is potentially around the corner (Brunyé et al., 2014).”

In the 1970's and 1980's, it was noted that event-related potentials (ERPs) could be generated in response to external or internal stimuli. Biofeedback of EEG activity enabled subjects to engage in intentional activities like moving an image on a television screen or a cursor on a computer monitor. With “P” standing for “electrical positivity” and “300” for the delay in milliseconds between stimulation and voltage change, the so-called P300 wave allowed neurotypical volunteers to spell words on a computer screen. In the clinical setting, microelectrodes inserted into specific brain areas began to be experimentally employed in the early 2000's with patients suffering from loss of limb control. The case of spinal cord injury patient Matt Nagle was described in *Wired* in 2005. Nagle, who learned how to control a computer cursor, was a participant in a clinical tried called “BrainGate.” Followed by BrainGate2, as reported in NRC (2009), brainstem stroke patient Cathy Hutchinson used a prosthetic arm to drink a bottle of coffee. These studies employed cables that tethered the patient-subject to brain signal-decoding computers, significantly limiting movements. In 2021, the BrainGate group announced successful experiments with an intracortical wireless BCI (an iBCI) with an external wireless transmitter.

Both the National Institutes of Health (NIH) and the Defense Advanced Research Projects Agency's (DARPA) Biological Technology Office (BTO) have committed to substantial investment in, *inter alia*, brain-computer interfaces connecting warfighters to computers through their brains. These neurotechnologies are a potential key to future US national defense, as well as a potential risk if developed by adversaries. More ambitious goals reach beyond simple EEG analysis and recording typical of implants and headsets to the use of AI to enhance BCI function is a central component of emerging military innovation. The BTO has described the ultimate goal of BCI as “BCI-AI fusion,” where AI and a human user communicate bidirectionally to share control over a task or system. This combination of human and artificial cognition is seen as a key strategic asset in future conflicts. In launching the new BTO program “Next-Generation Nonsurgical Neurotechnology (N),” Almondi noted that “DARPA is preparing for a future in which a combination of unmanned systems, artificial intelligence, and cyber operations may cause conflicts to play out on timelines that are too short for humans to effectively manage with current technology alone.” By connecting warfighters and decision makers to AI, rapid response to electronic and kinetic warfare can be managed using the skills humans and machines excel at, and keep a human in (or on) the loop in vital operations. In theory, the opportunities are remarkable. In the words of two IBM computer scientists, “[n]eurotech can interact with neurodata either invasively and directly through different kinds of surgical implants, like electrodes or devices implanted into or near neuronal tissues, or they can interact non-invasively and indirectly through wearable devices sitting on the surface of the skin…”.

There is already high-level attention among military planners to these possibilities for technologically mediated cognitive enhancement, not all of which appear in the first instance to be relevant to AI. Commercial EEG-detection neurotechnologies in headsets like Emotiv and NeuroSky have garnered public attention but are not AI-enabled. However, military planners are anticipating the convergence of headsets and AI. In 2017 a US Navy Special Operations commander called for the development of a non-invasive brain stimulation (NIBS) device that uses electrical stimulation to improve performance. A product of the company Halo Neuroscience, the Halo Sport Headset (based on electrical stimulation *via* tDCS) was designed to improve physical performance but was noted anecdotally also to improve cognition. It is reported to have been tested on Navy SEALs at five sites for cognitive enhancement, resulting in improved performance, as in the case of ameliorating the consequences of sleep-deprivation. Although a NIBS device is not in itself AI-enabled, like many other neurotechnologies it can be linked to an AI system to record and modulate neural activity, potentially improving the efficacy of the enhancer. Such “closed loop” AI-enabled systems can self-correct using feedback control to improve their devices' targeting and reliability. Nonetheless, if they modify cognition, even devices worn on the surface of the skin may be functionally equivalent to invasive devices.

### Current state of military brain enhancement and ethics

Brain enhancement experiments (including BCI as a prominent example) have attracted notice in the US in the form of expert advisory reports. Here we note several of those produced mainly by the National Academies of Science, Engineering and Medicine (NASEM), as these are most relevant to warfighter enhancements and neurotechnologies. Several US presidential advisory commissions have also issued reports that are relevant more generally to experiments involving warfighters. Some consensus has crystallized around an intuitive definition of enhancement in terms of a contrast with therapeutic interventions. In their report *Beyond Therapy* (2003), the President's Council on Bioethics articulated that consensus view:

“Therapy,” on this view as in common understanding, is the use of biotechnical power to treat individuals with known diseases, disabilities, or impairments, in an attempt to restore them to a normal state of health and fitness. “Enhancement,” by contrast, is the directed use of biotechnical power to alter, by direct intervention, not disease processes but the “normal” workings of the human body and psyche, to augment or improve their native capacities and performances^21^.

Like the President's Council and other authorities, we find the distinction of enhancement versus therapy the most useful rule-of-thumb.

Of more immediate interest is the Council's concern that “biotechnical power” could be used to modify the human psyche in particular, well “beyond therapy,” is what many find intuitively objectionable. Yet, as Lin et al. (2013) note in their research study on enhanced warfighters, “it is unclear how these objections would apply to the military context, e.g., whether they would be overcome by the special nature of military service and the exigencies of military operations….” Apart from the question of the acceptability of enhancement in the military setting in general, the acceptability of particular enhancements is a matter of perspective of different types of warfighters and their superiors, of their unit and third parties such as family members, of other military members, of civilians with whom they interact, of the government, and of the public and the nation. The history of modifying the human psyche “beyond therapy” is, moreover, arguably already common in many militaries in which the reluctance to kill other humans has been seen as a trait that needs to be trained out of warfighters (Evans and Hereth, forthcoming).

One of the few studies of its kind, the US National Academies report entitled *Opportunities in Neuroscience for Future Army Applications* (NRC, 2009) was an ambitious attempt to assess historical, ethical, and cultural issues for neuroscience in the army; neuropsychological testing in soldier selection, training, and learning; optimizing decision making; improving cognitive and behavioral performance (“hours of boredom and moments of terror”); neurotechnology opportunities like BCI; and long-term trends in research such as neural correlates for cultural differences in behavior. The same 2009 report described “in-helmet EEG for brain-machine interface” as a high-priority, medium-term (5–10-year) application opportunity. The report committee presciently emphasized that neither these kinds of opportunities, nor the points outlined in its 15 recommendations, would come to fruition without a single place in the Army to monitor potential neuroscience progress, evaluate potential applications and conduct the appropriate experimental research.

Perhaps surprisingly considering the subject of the report, although there is a section on the ethical issues raised by genetic screening of healthy persons, the report does not specifically address ethical issues about neurotechnologies beyond presupposing compliance with federal guidelines and regulations. It does raise the question of the applicability of research results derived from the usual volunteer subjects like undergraduate students, or even clinical patients, to a soldier population. Better surrogates might be high-performance athletes about whom there is extensive neuropsychological data. They may even be far superior subjects. When it comes to actual applications there are other challenges, including little knowledge of the candidate's psychology that may be relevant to their communication with other humans and to machines. In a chapter on neurotechnology opportunities, the report addresses issues like the physical load of any new device (not adding more than 1 kg to the helmet or 2 kg to the pack, not interfering with ballistic protection or helmet stability or freedom of head movement), field-deployable markers of neural state, EEG-based computer interfaces, haptic feedback for virtual reality, and augmented reality technologies, among others.

## Ethical considerations for AI-enabled neurotechnology experimental research

Emerging AI-enabled neurotechnologies that may ultimately be operationally deployed present opportunities for warfighting and novel challenges to ethical standards for research and development involving warfighters. “Neuroenhancement” marries such life sciences as neurology, pharmacology, genetics, and psychology with long-time soldiering attributes that include endurance, speed, intelligence-gathering, targeting, and training, none of which are medical conditions. As with any military technology, neuroenhancement products move slowly from research and development to field use.

At the research stage, ethical criteria require clinical investigators to establish the value and necessity of their proposed research, demonstrate a favorable cost/benefit ratio, utilize valid scientific methods, and protect research subjects' rights and welfare (Emanuel et al., 2000). Chief among research subjects' rights is informed consent that healthy volunteer research subjects must provide. Informed consent respects agents' dignity and right to self-determination by affording research subjects the information they require to weigh the costs and benefits of participating in medical research. Given the checkered history of military medical experimentation (Faden et al., 1995; Siegel-Itzkovich, 2009); however, the rules and regulations for clinical research among service personnel include special protections.

Following non-military clinical research protocols for vulnerable populations, military organizations in the US and Europe institute provisions to protect military research subjects' rights. Military officials understand that formal expressions of consent do not guarantee its respect. Although soldiers sign consent forms, problems arise because of rank disparity, fears of offending one's superiors, and/or peer pressure, which may undermine informed consent when soldiers are asked to participate in medical experiments (European Parliament, 2014, para. 31). As a result, additional regulations oversee clinical research and protect research subjects from coercion. The importance of voluntary consent is especially strong in cases where medical enhancements are *irreversible* (Davidovic and Crowell, 2022).

To safeguard voluntary consent among service members, The DoD's *Human Subjects Protection Regulatory Requirements* (Department of Defense, 2019, also: 32CFR219, “Protection of Human Subjects,” and US Department of Defense Instruction 3216.02, 2018, 45 CFR 46, 2019) forbids the involvement of superior officers during the solicitation of research subjects and demands informed consent, medical supervision, the right to end an experiment, and the employment of an independent ombudsman or research monitor to oversee recruitment and experimentation [Department of Defense (DoD), 2011, p. 24–25]. British military officials, like their American counterparts, appoint an independent medical officer (IMO) to monitor the health, safety, and wellbeing of the participants (UK Ministry of Defense, 2020, p. 8; Linton, 2008).

To ensure that investigators meet statutory and ethical guidelines, independent and multidisciplinary Institutional Review Boards (IRB) in the United States (Department of Defense (DoD), 2011, p. 11–29), and Ministry of Defense Research Ethics Committees (MODREC) in the United Kingdom (UK Ministry of Defense, 2020), oversee research approval and compliance. Research oversight is complicated and time-consuming. Charged with what British officials term “proportionate scrutiny” (UK Ministry of Defense, 2020, para. 2–5), committee members seek a balance between outcomes and rights. Outcomes comprise benefits net of cost. Rights speak to respect for dignity and autonomous decision-making, informed consent, and acceptable risk.

These safeguards, however, are only part of the picture. They formally ensure informed consent, but researchers must provide adequate data to give substance to the right. Notice how emerging technologies pose medical risks for healthy research subjects while, at the same time, the operational goals of enhancement, that is, mission success, are entirely military. Therefore, ethically sustainable neuro-enhancement military research requires investigators to address two questions simultaneously so they may attain critical military goals while protecting research subjects' rights:

1. Is the proposed enhancement technology medically and militarily necessary?
2. Do the medical and military risks outweigh their benefits?

The following sections consider each of these questions in turn.

### Medical necessity: What medical advantages does clinical research provide?

The overriding goal of any therapeutic clinical study is *medical* necessity. Investigators must demonstrate the likelihood that a new technology or medical procedure will not only effectively save lives or improve their quality but is also necessary. “Necessary” means that no other technology or procedure will attain the same outcome at a lower cost. There are no grounds to research a costly medical device, for example, if it is only as effective as a much less expensive existing technology. Therefore, it would be egregiously unethical to pursue unnecessary human research. However, non-therapeutic enhancements are neither curative nor rehabilitative. They do not save or improve the lives of the sick or injured. What medical benefit, then, do they provide warfighters? In what way are they *medically* necessary? One answer is that they are not. Enhancement provides research subjects with no medical benefits. Is conducting such research, therefore, ethically permissible?

There are two ways to address this objection. In one respect, enhancement research offers experimental subjects a personal benefit. As enhancement technologies push beyond normal baseline capabilities, they can boost a person's memory, sensory acuity, or targeting accuracy and, in this way, improve some warfighters' chance of survival. While surviving one's occupation is immensely valuable to the survivor, it is nonetheless largely instrumental in a military context. By optimizing warfighter performance, successful enhancement improves the prospect of mission success. As it does, mission, not medical, success assumes the metric for measuring the necessity of cognitive enhancement research.

In saying this, we do not mean to assert that every warfighter enhancement *directly* benefits the enhanced individual. It probably does not. However, this leaves open the possibility that successful warfighter enhancements—i.e., enhancements that support strategic dominance and actualize military objectives—*indirectly* benefit enhanced individuals. As an analogy, consider vaccinations. As Jason Brennan observes,

[T]he problem is that individuals as individuals make little difference. If everyone in the world were vaccinated except for Andy and Betty, Andy and Betty would pose no real threat to each other. Instead, vaccination presents a collective action problem, in which individuals as individuals are unimportant. […] In general, individual decisions to vaccinate or not have negligible effects on others. What matters is what *most* people do, not what individuals do (Brennan, 2018, p. 39, 40).

When enough individuals are vaccinated, herd immunity is achieved. Herd immunity benefits *the herd*, a group of individuals, and by extension benefits *most members* of the herd. In a similar way, warfighter enhancements provide a kind of ‘herd immunity' that protects against military failure, which in turn protects warfighters as a group and, therefore, *most individual* warfighters. Thus, the relevant kind of ‘medical necessity' entailed by military necessity is equivalent to the kind of “medical necessity” entailed by public health necessity, as illustrated in the case of vaccinations.

Mission success, however, is fundamentally a *military*, not a *medical*, benefit that researchers and institutional review boards (IRBs) must weigh against a medical risk as they evaluate a project's feasibility. Like individual soldiers, IRBs face a utility calculation of incommensurable values: medical risks and military benefits. In practice, however, IRBs may resist this balancing act and instead search out individual medical or personal benefits, such as resiliency or language proficiency, that a research subject may acquire from participating in an experiment. But these personal advantages cannot be the determinative counterweight to individual risk in cognitive enhancement research. An enhancement technology that optimizes target selection, for example, may offer no discernable advantage to the research subject. In this situation, military benefits alone offset the medical risks of experimentation and provide the rationale for IRB ethics approval.

In this environment, researchers must proceed differently when conducting experimental studies than in clinical studies. They must convincingly argue that their proposed technology, a BCI, for example, is militarily necessary in the same way that therapeutic interventions are medically necessary. This requirement mirrors clinical guidelines that remind researchers, “because a normal healthy subject does not directly benefit from the study, the risk-benefit analysis must focus strongly on *the importance of the knowledge to be gained*” (e.g., Cornell University Office of Research Integrity, emphasis added). In this case, the knowledge gained is medical so that healthy research subjects must satisfy themselves that the greater good they serve (important medical knowledge) offsets the personal risk they incur during experimentation. In contrast, the critical knowledge provided by neuro-enhancement experimentation is primarily military. As a result, research subjects balance the medical risks of enhancement against its military benefits, a dramatically different sort of calculus to assess necessity.

### Military necessity: What military advantages does enhancement research offer?

A recent RAND report (Binnendijk et al., 2020), *Brain Computer Interfaces: US Military Applications and Implications*, turns to military and technical specialists to evaluate brain-computer interfaces during urban operations in asymmetric war (p. 6). Using BCI as their test case, they asked: “which [BCI] capabilities [e.g., communication management, weapons control, enhancement cognitive or physical performance and training] were seen as more *useful* to support complex ground operations (emphasis added).” While the results certainly contribute to the BCI debate, the experimental design overlooks the question of necessity. Usefulness is not necessity. Asked to choose among seven BCI technologies, respondents were not asked to compare these to existing technologies that might improve training, weapons control, or communication. And while they may have been useful, there was no way to know if they were necessary and therefore, viable candidates for human research.

More critically, the RAND study's experimental design focused on a narrow range of counterinsurgency (COIN) operations: clearing a building of insurgents and evacuating wounded warfighters. This choice of cases raises two questions. First, how central are these tactical operations to asymmetric war? Second, is asymmetric war the paradigm we should use for evaluating BCI? One of us has argued, for example, that contemporary counterinsurgency warfare pushed well-beyond the kind of urban warfare described in the RAND report to include drone attacks, cyber and information warfare and, above all, population-centered counterinsurgency and public diplomacy to win “hearts and minds (Gross, 2021, p. 181–203).” Among the neuro-enhanced skills required for COIN are language acquisition, cultural knowledge, and conflict management. The ideal soldier in modern asymmetric war may not be “a super-empowered soldier able to perform solo missions and transmit data back to headquarters” (Malet, 2015, p. 3); also (Galliott and Lotz, 2017), but one closer to Kaurin's description of a “Guardian.” The Guardian embodies “soft” warfighting skills that attend to the needs of the weak and vulnerable, resolves issues without the use of force, pays attention to “culture, language and politics,” and displays adaptability (Kaurin, 2014, p. 89–90).

Asymmetric war, moreover, is not the only game in town. On the one hand, NATO nations may intervene in conventional set-piece warfare as it currently wracks Ukraine. On the other, the West may veer toward near-peer confrontations with China or Russia or confront nuclear threats from Iran and North Korea. In the latter instances, emphasis shifts from traditional warfighting concerns of offsetting troop strength and military assets to offsetting an adversary's rapid technological advancements. New technologies include advanced computing, “big data” analytics, artificial intelligence, autonomy, robotics, directed energy, hypersonics, and biotechnology [Department of Defense (DoD), 2018]. In the words of one group of Chinese neuroscientists, “Artificial intelligence (AI), which can advance the analysis and decoding of neural activity, has turbocharged the field of BCI” (Zhang et al., 2020).

With the “turbocharging” of BCI by AI in mind and considering the scenarios of contemporary and near-term warfare one must ask where and how neurotechnologies like BCI are useful and necessary in these contexts. What is this technology's highest and best use? While implantable iBCI may enable a generation of bionic warfighters, their role in contemporary and future warfare remains unsubstantiated and, perhaps, marginal. In contrast, EEG-based eBCI significantly improve training and learning by offering feedback loops to evaluate data and monitor performance by a human operator. Similarly, non-invasive nerve stimulation devices such as earbud electrodes enable targeted neuroplasticity training (TNT) to accelerate language acquisition, acculturation, and intelligence analysis to facilitate successful population-centered COIN. eBCI and other TNT neuro-technologies help operators organize information flows to permit fast-moving threat and target identification (Naufel et al., 2020). In these ways, eBCI do not enhance the killing capabilities that some iBCI may offer warfighters. Instead, they can improve the quality of the intelligence warfighters receive while enhancing the soft skills required to attend to the needs of the local population.

Evaluating military necessity at the research stage is a speculative but essential endeavor that should integrate military analysts into the preparation of clinical studies. But the absence of any sustained discussion of military necessity is glaring. Nevertheless, many researchers avoid the discussion of military benefits altogether or only offer perfunctory details. A 2019 consent form from the US Army Aeromedical Research Laboratory, for example, makes short shrift of potential military benefits of anti-fatigue agents. It simply advises potential research subjects, “*Your participation will contribute to the medical knowledge and scientific investigation of possible uses for these medications in a military operational setting*.” Under UK Ministry of Defense Research Ethics Committee (MODREC) guidelines entitled, “Participant Involvement: Risks, Requirements and Benefits,” Paragraph 17h instructs researchers to “*describe any expected benefits to the research participant (if none, state none)*.” “None” only makes sense if the expected benefits are solely medical. In neither example do researchers “focus strongly on the knowledge to be gained” from experimentation. To do so will inevitably draw military policymakers and ethicists into enhancement research.

To provide fully informed and voluntary consent, research subjects must also contend with military and medical *risks*. Medical risks may be physiological and/or psychological and may render some technologies that require surgical implantation, for example, unsustainable. Here, issues related to the vulnerability of specific populations come into play. Military risk is both technological and organizational. The former includes vulnerability to hacking and data theft, while the latter raises concerns about disseminating and protecting data among the many interested stakeholders in a military organization.

### Medical risks

Surgically implanted brain-computer interfaces pose significant medical risks leading DARPA to reject surgically invasive enhancement techniques:

Due to the inherent risks of surgery, these technologies have so far been limited to use by volunteers with clinical need. For the military's prima-rily able- bodied population to benefit from neurotechnology, *non-surgical interfaces are required*. Teams are pursuing a range of approaches that use optics, acoustics, and electromagnetics to record neural activity and/ or send signals back to the brain at high speed and resolution. The re-search is split between two tracks. Teams are pursuing either completely non- invasive interfaces that are entirely external to the body or minutely invasive interface systems that include nanotransducers that can be tempo-rarily and non-surgically delivered to the brain to improve signal resolution [Defense Advanced Research Projects Agency (DARPA)., 2019, emphasis added].

Some observers concur: “To effectively implement BCI systems… for enabling *efficient performance by healthy users*,” write Miranda et al. (2015, p. 64), “there exists a need for the development of subcutaneous and *fully non-invasive* neural interfaces that are both portable and capable of recording activity from cortical and deep brain structures at high spatial and temporal resolution (emphasis added).” However, others draw a line between research and deployment. “Despite the high accuracy and optimal signal fidelity [of intracortical electrodes],” write Portillo-Lara et al. (2021, p. 3), “the risks associated with the surgical procedures largely restrict their use outside well-controlled laboratory and clinical environments.” Similarly, “greater risk may be tolerable for the restorative technologies… in the clinical domains, but could be less ethically justifiable for the performance benefits for healthy individuals” (Naufel and Klein, 2020, p. 5).

Rejections of high-risk, implantable neurotechnologies for healthy individuals are *de rigueur* but not always accompanied by convincing ethical arguments. Despite legitimate apprehension about coercion and undue influence that comes from “institutional or hierarchical dependency (European Parliament, 2014, para. 31),” military personnel are not a vulnerable population on par with minors, prisoners, or the economically disadvantaged, as some suggest (McManus et al., 2007; Parasidis, 2016). Service personnel do not lack sound decision-making capacity or suffer from socially inflicted disabilities. There are no a priori reasons that render service personnel incapable of making informed choices about their participation in medical research or willingness to accept these risks if counterbalanced by military or, to a lesser extent, medical benefits.

Researchers may also reject invasive neuroenhancements because they believe the risk is too high or insufficiently known (e.g., Nijboer et al., 2013, p. 553). Naufel and Klein (2020, p. 2) cite a 20–40% risk of surgical complications and 24–50% risk of hardware complications. Additionally, researchers and funding agencies may think alternative semi-invasive or non-invasive neurotechnologies are adequate for military purposes. Whether implantable technologies are *necessary* is a logically prior question that demands an answer before considering surgical risks. Until it is, there is no *prima facie* reason to reject invasive technologies.

bib44 If implantable BCI pose the danger of surgery and interface maintenance, eBCI are not entirely without risk. Researchers note unknown psychological risks affecting personality, memory, and BCI dependence (Vlek et al., 2012; Kögel et al., 2019; National Academies of Sciences Engineering Medicine, 2021, p. 41, 50). Incorporating AI in BCI adds additional unpredictability and risk. Unlike traditional BCI, whose functions may be static, a self-correcting AI can dynamically adapt how it operates. As a result, additional risks may accumulate as research subjects interact with BCI and AI-enabled BCI react and adapt to stimuli.

Nevertheless, evaluating such risks is integral to the research project. As such, research subjects require a good-faith assessment of these risks and the means to mitigate them should adverse psychological effects or unpredicted AI adaptations surface during or after the experiment. It is challenging to present potential psychological or AI-related risks to research subjects when their full extent is unknown until the trial concludes. Phase 1 drug trials, for example, investigate toxicity. As such, research subjects cannot receive but scant information about potential risks. However, buoyed by optimism and “therapeutic misestimation” that exaggerates a trial's benefits, critically ill research subjects often discount the risks and consent to experimental treatment (Miller and Joffe, 2013; Halpern et al., 2019). However, military research subjects for cognitive enhancement are not ill. There are few or no medical benefits to excite sufficient sanguinity to offset thinly demonstrable risks. As a result, non-therapeutic researchers operate under stricter conditions than clinical researchers. We can only speculate about the psychological effects of BCI (personality changes, memory disruptions, or BCI dependence) and the additional risks of AI-enabled BCI because one research goal is to study these effects. But to obtain fully informed and voluntary consent, research subjects also need additional data about technological and institutional risk.

### Risks: Technological and institutional risks to privacy and confidentiality

Technological risks comprise BCI hacking that may put personal information in hostile hands. Institutional risks come when myriad stakeholders claim privileged information, including related agencies, the scientific community, pharmaceutical companies, and perhaps, allied nations. This coterie of stakeholders is not unique in military medicine, where patients have limited rights to their personal medical data (Gross, 2021). Technological and institutional risks impinge upon privacy and confidentiality, two fundamental rights of research subjects.

Privacy and confidentiality are closely related. Privacy is a subsidiary right of personal self-determination: the right to keep information close and release only what one wants others to know about oneself (Bok, 1989, p. 120). Confidentiality is a duty imposed on others to guard another's private information until that person authorizes its disclosure. The right to privacy and the duty of confidentiality ensure self-esteem, job security, and social status that the release of personal information may jeopardize. In medicine, respect for privacy preserves the trust necessary for practitioners to tend patients successfully and for researchers to maintain the trust they need to conduct clinical trials. Usually, privacy and confidentiality are straightforward. Patients disclose information so medical practitioners can provide proper care. Beyond that, it is nobody's business.

Novel risks to autonomy are also raised by the prospect of neurointerventions. For example, deep-brain stimulation (DBS) applied therapeutically to Parkinson's patients has undermined patients' sense of personal authenticity and enhanced their sense of alienation, leading some (e.g., Kraemer, 2013) to conclude that DBS poses serious risks for autonomy, and others to propose non-individualistic conceptions of autonomy (Lee, 2021). Indeed, some scholars contend that theoretical neurointerventions provide a basis for ethical theorizing about the nature of autonomy (Zuk and Lázaro-Muñoz, 2021). By contrast, other scholars like Douglas (2022) argue that just as “nudges” can treat their targets as rational agents, so too can non-consensual neurointerventions. Plausibly, the possibility of treating one's targets as rational agents entails the possible retention of their autonomy, such that even *non-consensual* neurointerventions might respect autonomy (cf. Gillett, 2009). Even more controversial is Pugh (2014) claim that some neurointerventions, such as those that reduce impulsivity, can *enhance* patient autonomy (cf. Fleishmann and Kaliski, 2017).

Clinical research is bound by weaker rules of privacy than medical practice is. For example, research subjects may be required to share large chunks of anonymized data as part of the experimental research (Malin et al., 2010). In addition, AI-assisted enhancement research may further attenuate privacy, thereby requiring researchers to provide healthy research subjects with answers to the following questions:

1. Data attributes: What kind of data and in what format do BCI record? What personal or ancillary information do the data reveal?
2. Data accessibility and sharing: Who has access to the data? What agreements are there for data sharing? Who can potentially read this data?
3. Data protection: How are the data protected? Where are the data stored during and after the experiment? Are the experimental BCI vulnerable to hacking as some fear?

The answers to these questions are the subject of research itself. Most iBCI use intracortical devices to measure neuron activation potential in particular brain regions, often on the level of individual neurons. eBCI tends to use fMRI or EEG signals. Both signals measure activation potential, usually across large segments or the whole of the brain. Typically, voltages or activation potentials correspond to particular mental states. These are neural correlates the machine receives as the basis for action. As a result, there are concerns regarding invasions of privacy, unauthorized access to confidential information and hacking. In response, data management plans, software fault tree testing, and red teams (that try to hack the machine on behalf of the manufacturer) address these concerns. They are integral to a research ethics protocol (Denning et al., 2009).

Finally, while the technological risks associated with utilizing AI are broad and cannot be adequately summarized in this paper, we would be remiss if we failed to mention a few crucial areas of concern. First, AI has well-known racial (Kostick-Quenet et al., 2022)^6^, gender (Wellner and Rothman, 2020; Waelen and Wieczorek, 2022), and disability biases (Tilmes, 2022). These algorithmic biases undermine the permissibility of unthinking reliance on purportedly “unbiased” AI. Second, AI decision-making is notoriously opaque – its decisions are made, as multiple scholars have described it, in an “algorithmic black box” (Hollanek, 2020; von Eschenbach, 2021). Despite occasional optimism about rendering AI decision-making transparent (e.g., Mishra, 2021), most scholars remain concerned about the effects of biased AI used for medical purposes. Among these are concerns that biased AI will reduce persons to mere data (Sparrow and Hatherley, 2019), that AI might impermissibly (and invisibly) incorporate economic data in its rationing recommendations (Sparrow and Hatherley, 2020; Braun et al., 2021), and that AI will rely upon other value-laden considerations (Ratti and Graves, 2022). Again, this is merely a sampling of the technological risks associated with AI. The risks extend well-beyond algorithmic bias. Yet these risks must be considered when evaluating the permissibility of AI-enabled warfighter enhancements.

## Moving forward: Sustainable research ethics for neuroenhancement military research

Research protocols for *therapeutic* neurotechnologies draw attention to respect for autonomy, informed consent and self-determination, the right to privacy and confidentiality, and constant concern for the welfare of subjects, their community, and end-users (Girling et al., 2017; Pham et al., 2018). To maintain the same respect for the rights of healthy research subjects who participate in *non-therapeutic* military neuroenhancement research demands attention to a full array of unique military and medical costs and benefits. Therefore, any sustainable ethics protocol for non-therapeutic neuro-enhancement military research must closely note military and medical risks and benefits to adequately protect research subjects' rights. To date, most researchers fail to fully account for a novel technology's expected military benefits, sometimes over-compensate for military research subjects' vulnerability, fail to consider the technological and institutional risks to privacy and confidentiality and overlook the intricacy of balancing often incommensurable apples (medical risks) and oranges (military necessity).

Research subjects, therefore, adopt a utility calculus common in the military that positions personal risk against collective benefits. By taking stock of national or military interests, they may accept considerable personal risk if the military benefits accruing to their political commonwealth are significant. Attention to military necessity and collective social interests at the expense of individual wellbeing is not foreign to military medical ethics. The US Army Medical Department (AMEDD) *Emergency War Surgery* (Cubano, 2018), for example, reminds its per-sonnel: “the ultimate goals of combat medicine are the return of the greatest possible number of warfighters to combat and the preservation of life, limb, and eyesight. Commitment of resources should be decided first based on the mission and immediate tactical situation *and then* by medical necessity, irrespective of a casualty's national or combatant status” (Cubano, 2018, p. 24, emphasis added; cf. JP 4- 02, 2001: II- 1; 2006:ix). And while this provision applies to therapeutic care, it informs research priorities as well.

More data and greater sensitivity drive the way forward. AI-enabled neuroenhancement offers tremendous possibilities for military use to improve warfighting capabilities, reduce service members' exposure to life-threatening danger and meet emerging threats. But sensitive to research subjects' rights, investigators must spell out the military advantages in far greater detail while IRBs supervise compliance. Although data collected from large numbers of healthy, young warfighters may turn out to be instructive for medical science, no military medical research protocol should content itself with simply telling subjects that they are taking significant risks for *medical knowledge …in a military operational setting*. Non-therapeutic military neuro-enhancement research protocols also cannot suffice with compiling medical risks alone. Moreover, there are ethically relevant differences between clinical research and non-therapeutic military medical research which draws in vested stakeholders and parties with access to information. Unlike clinical medical research, military medical research is likely to attract hostile parties who may put subjects at considerable risk. In this way, neuro-enhanced soldiers share the attributes of newly developed weapons, and their nations must acknowledge the danger they face and protect them accordingly.

Despite two decades of speculation about the prospects for neuro-enhancement amid the convergence of BCI and AI, an array of ethical issues that remain to be sorted out have been an obstacle to the systematic investigation of operational potential. To fill the lacunae of basic BCI/AI research, we have suggested a comprehensive and critical analysis of military necessity comparable to medical necessity. Medical necessity recounts the overwhelming advantage a new technology, intervention, or drug will offer individual patients and society. Military necessity must do the same for neurotechnologies designed to enhance warfighter performance while taking account of the conditions necessary to obtain fully informed and voluntary consent.

## Data availability statement

The original contributions presented in the study are included in the article/supplementary material, further inquiries can be directed to the corresponding author.

## Author contributions

All authors listed have made a substantial, direct, and intellectual contribution to the work and approved it for publication.

## Funding

This paper was funded under a grant by the U.S. Air Force Office of Scientific Research, award number FA9550-21–1-0142.

## Conflict of interest

The authors declare that the research was conducted in the absence of any commercial or financial relationships that could be construed as a potential conflict of interest.

## Publisher's note

All claims expressed in this article are solely those of the authors and do not necessarily represent those of their affiliated organizations, or those of the publisher, the editors and the reviewers. Any product that may be evaluated in this article, or claim that may be made by its manufacturer, is not guaranteed or endorsed by the publisher.

## Footnotes

^1^See for example Erik J. Larson, *The Myth of Artificial Intelligence: Why Computers Can't Think the Way We Do* (Cambridge, Mass: Harvard University Press, 2021).

^2^For nonmilitary readers, “offsets” refer to the balance of force between nations, usually in great power conflict. The first offset in US doctrine is nuclear deterrence, and the second is stealth and precision guided munitions to counter larger hostile numerical forces. So, the “third offset” often gets used to describe some emerging set of technologies that will shift the balance of power, and usually (though not always) this is AI/autonomous systems.

^3^For efficiency in this paper BCI will be assumed to include human computer interface (HCI) with specificity in the context of the discussion.

^4^Elon Musk's Neuralink is the best known of these companies but there are other startups in this space with different approaches, especially in the ways that neural activity is recorded. Among the more innovative approaches, Stentrode introduces stents in blood vessels rather than some form of invasive bioelectrode or surface sensor.

^5^Perhaps there was something in the water at that time in Los Angeles: Only a year later the first BCI that came to the attention of many Baby Boomers was the one featured in a 1966 Star Trek episode in which a severely brain injured Captain Christopher Pike uses such a system to communicate, but in this primitive approach the user was limited to one signal for “yes” and two signals for “no”.

^6^Interestingly, the use of AI – in particular, the use of avatars – can *reduce* implicit racial bias (Peck et al., 2013). Thus, the use of even racially biased AI could theoretically mitigate racial biases in human users.
